# The proliferative effects of *Pyropia yezoensis* peptide on IEC-6 cells are mediated through the epidermal growth factor receptor signaling pathway

**DOI:** 10.3892/ijmm.2015.2111

**Published:** 2015-02-26

**Authors:** MIN-KYEONG LEE, IN-HYE KIM, YOUN-HEE CHOI, JEONG-WOOK CHOI, YOUNG-MIN KIM, TAEK-JEONG NAM

**Affiliations:** 1 Department of Food Science and Nutrition, Pukyong National University, Busan 608-737, Republic of Korea; 2 Institute of Fisheries Science, Pukyong National University, Busan 619-911, Republic of Korea

**Keywords:** *Pyropia yezoensis*, peptide, proliferation, epidermal growth factor receptor, signaling pathway, cell cycle

## Abstract

For a number of years, seaweed has been used as a functional food in Asian countries, particularly in Korea, Japan and China. *Pyropia yezoensis* is a marine red alga that has potentially beneficial biological activities. In this study, we examined the mechanisms through which a *Pyropia yezoensis* peptide [PYP1 (1–20)] induces the proliferation of IEC-6 cells, a rat intestinal epithelial cell line, and the involvement of the epidermal growth factor receptor (EGFR) signaling pathway. First, cell viability assay revealed that PYP1 (1–20) induced cell proliferation in a concentration-dependent manner. Subsequently, we examined the mechanisms responsible for this induction of proliferation induced by PYP1 (1–20). EGFR is widely expressed in mammalian epithelial tissues, and the binding of this ligand affects a variety of cell physiological parameters, such as cell growth and proliferation. PYP1 (1–20) increased the expression of EGFR, Shc, growth factor receptor-bound protein 2 (Grb2) and son of sevenless (SOS). EGFR also induced the activation of the Ras signaling pathway through Raf, MEK and extracellular signal-regulated kinase (ERK) phosphorylation. In addition, cell cycle analysis revealed the expression of cell cycle-related proteins. The results demonstrated an increased number of cells in the G1 phase and an enhanced cell proliferation. In addition, the upregulation of cyclin D, cyclin E, Cdk2, Cdk4 and Cdk6 was observed accompanied by a decreased in p21 and p27 expression. These findings suggest that PYP1 (1–20) stimulates the proliferation of rat IEC-6 cells by activating the EGFR signaling pathway. Therefore, PYP1 (1–20) may be a potential source for the development of bio-functional foods which promotes the proliferation of intestinal epithelial cells.

## Introduction

Seaweed is widely consumed throughout Asian countries, such as China, Japan, Korea, Vietnam, Indonesia, the Philippines and Hawaii, as a natural medicinal and food source (1). Seaweed contains a variety of nutrients, such as carbohydrates, vitamins, minerals, fatty acids, dietary fiber, amino acids, iodine and essential polysaccharides. Research on the chemical and nutritional composition of seaweed has revealed that it has antioxidant, antitumor and antibacterial activities (2,3). The red seaweed, *Pyropia yezoensis*, is an economically important seaweed in Asian countries, such as China, Japan and Korea. *Pyropia yezoensis* is an important source of physiologically active substances that contain 25–50% protein and 25–40% carbohydrates based on dry matter weight (4). *Pyropia yezoensis* has been shown to exert biological effects, such as antitumor, anti-fatigue, blood pressure-reducing, anti-inflammatory, antioxidant and hepatoprotective effects (5,6). In a previous study, we examined a peptide from *Pyropia yezoensis* which stimulates the proliferation of IEC-6 cells by activating the insulin-like growth factor I receptor signaling pathway (7). In the present study, we examined the effects of a *Pyropia yezoensis* peptide [PYP1 (1–20)] on cell proliferation and related EGFR signaling pathways in IEC-6 cells, a rat intestinal epithelial cell line.

Cell proliferation is induced through intracellular signal transduction mediated by receptor tyrosine kinases (RTKs), such as epidermal growth factor receptor (EGFR) (8). RTKs are cell membrane receptors for growth factors and other extracellular ligands. RTKs mediate cellular tyrosine phosphorylation and regulate intracellular signaling pathways, such as those involved in cell migration, differentiation and proliferation (9–11). RTKs of the EGFR family mediate essential cellular functions, including the regulation of cellular proliferation, growth, survival, migration, differentiation and development in normal and pathological states (12,13). The binding of EGF to EGFR initiates a number of molecular events. The EGF-EGFR molecular interaction activates growth-promoting signals primarily through the activation of Ras, leading to activation of the Ras/Raf/mitogen-activated protein kinase (MAPK) and phosphoinositide 3-kinase (PI3K)/Akt pathways, as well as many others (14,15). EGFR is stimulated by the guanine nucleotide-exchange factor, son of sevenless (SOS)-growth factor receptor bound protein 2 (Grb2) complex. This activation of EGFR leads to the activation of Ras. The Src homology 2 (SH2) domain of Grb2 binds to autophosphorylation sites of EGFR, including Y1068, and several other receptors. The SH3 domains of Grb2 bind to the proline-rich C-terminal domain of SOS, a guanine nucleotide exchange factor (GEF) for Ras. In this way, the SOS-Grb2 interaction plays a critical role in regulating the activation of Ras (16,17). The small guanosine triphosphatase protein Ras/MAPK signaling pathway is essential for the regulation of a variety of biological processes, such as cell growth, cell cycle, cell proliferation and cell senescence, all of which are important for normal development (18). It consists of a core module of three kinases comprising Raf, MEK and extracellular signal-regulated kinase (ERK) that transmit signals downstream of the small GTPase, Ras. GTP-loaded Ras triggers the sequential activation of Raf, MEK and ERK to promote cell survival and various cellular functions (19,20). Therefore, EGFR and related proteins are attractive targets affecting cell proliferation.

The present study was carried out to confirm that PYP1 (1–20) promotes the proliferation of IEC-6 cells and to determine the molecular mechanisms responsible for its proliferative effects by investigating the involvement of the EGFR signaling pathway. Our findings suggest that PYP1 (1–20) affects EGFR-induced cell proliferation, a potential factor in intestinal epithelial cell protection.

## Materials and methods

### Pyropia yezoensis peptide synthesis

The N-terminal 20 residues of PYP1 (1–20) (A-L-E-G-G-K-S-S-G-G-G-E-A-T-R-D-P-E-P-T), designated as PYP1 (1–20), were synthesized by Peptron (Daejeon, Korea) (7,21). The purification of PYP1 (1–20) was performed using a Shimadzu Prominence high-performance liquid chromatography (HPLC) apparatus and the software package Class-VP, 6.14 (Shimadzu, Kyoto, Japan), with a C18 column (Shiseido Capcell Pak; Shiseido, Tokyo, Japan) in 0.1% TFA/water, a gradient of 10–70% acetonitrile in 0.1% TFA, a flow rate of 1 ml/min, and UV detection at 220 nm. The molecular mass of PYP1 (1–20) was confirmed to be 1,916 Da (matched with the sequence mass) by mass spectrometric analysis (HP 1100 Series LC/MSD) (21).

### Cell culture

The IEC-6 rat small intestinal epithelial cells (ATCC CRL-1592; ATCC, Manassas, VA, USA) were cultured in Dulbecco’s modified Eagle’s medium (DMEM) supplemented with 10% fetal bovine serum (FBS; HyClone, Inc., South Logan, UT, USA), 100 U/ml penicillin and 100 mg/ml streptomycin, at a temperature of 37°C in a humidified atmosphere of 5% CO_2_. The cells were cultured to 60% confluence in 100-mm dishes. The medium was replaced every 2 days.

### MTS assay

The effects of various PYP1 (1–20) concentrations on cell proliferation were determined colorimetrically after 24 h using the 3-(4,5-dimethylthiazol-2-yl)-5-(3-carboxymethoxyphenyl)-2-(4-sulfonyl)-2H-tetrazolium (MTS) assay with the Cell Titer 96 Aqueous One Solution reagent (Promega, Madison, WI, USA). The cells were seeded in 96-well plates at 1×10^4^ cells/well. After 24 h of incubation, the cells were maintained in serum-free medium (SFM) for 4 h. The medium was replaced with fresh SFM containing PYP1 (1–20), and the cells were incubated for an additional 24 h. The cells were exposed to MTS assay solution at 37°C for 30 min, and the optical density at 490 nm was measured using a microplate reader. The OD_490_ values of the control cells were designated as 100%.

### Western blot analysis

To prepare whole cell extracts, the IEC-6 cells in 100-mm dishes were cultured to 50–60% confluence and then incubated in SFM for 4 h. Fresh SFM containing PYP1 (1–20) (125, 250, 500 and 1,000 ng/ml) was added to the cells and incubated another 24 h, after which the cells were washed with phosphate-buffered saline (PBS) and suspended in extraction buffer (50 mM Tris-HCl, pH 7.4, 150 mM NaCl, 0.25% Na-deoxycholate, 1% NP-40 and 1 mM EGTA) containing protease inhibitors (1 mM Na_3_VO_4_, 1 *μ*g/ml aprotinin, 1 *μ*g/ml pepstatin, 1 *μ*g/ml leupeptin, 1 mM NaF and 1 mM PMSF) on ice. The extracts were centrifuged at 14,000 rpm for 10 min, and the supernatant was used in western blot analysis. Boiling sample buffer (30 *μ*g) was added to the total cell lysate, and the samples were boiled for 10 min at 100°C. Proteins were separated by 7.5–12.5% sodium dodecyl sulfate-polyacrylamide gel electrophoresis (SDS-PAGE) and transferred onto polyvinylidene fluoride membranes (Millipore, Billerica, MA, USA). The membranes were blocked for 1 h 40 min at room temperature in blocking buffer [1% bovine serum albumin (BSA) in TBS-T]. The blots were probed with primary antibodies [p-EGFR (sc-12351), EGFR (sc-03), Shc (sc-1695), Grb2 (sc-255), SOS (sc-259), Ras (#3965), Raf (sc-227), MEK (sc-219), p-ERK (sc-7383), ERK (sc-94), Cyclin D1 (sc-753), Cyclin E (sc-481), Cdk2 (sc-163), Cdk4 (sc-601), Cdk6 (sc-177), pRb (sc-16670), p21 (sc-397), p27 (sc-528), GAPDH (sc-25778) (1:1,000 and 1:2,000 in 1% BSA/TBS-T)] overnight at 4°C. The membranes were then washed twice for 15 min in TBS-T. The secondary antibody was a horseradish peroxidase (HRP)-conjugated goat anti-mouse or rabbit antibody [goat anti-mouse IgG-HRP (sc-2031), goat anti-rat IgG-HRP (sc-2032) (1:10,000 in 1% BSA/TBS-T)]. Signal bands were detected using an enhanced chemiluminescence (ECL) western blotting kit (Thermo Fisher Scientific, Inc., Rockford, IL, USA).

### Reverse transcription-polymerase chain reaction (RT-PCR)

The mRNA expression levels of specific genes were evaluated by RT-PCR (22). The IEC-6 cells were seeded into 100-mm dishes at a density of 2x10^4^ cells/well and cultured for 24 h, after which the medium was replaced with SFM containing PYP1 (1–20) (125, 250, 500 and 1,000 ng/ml) for 24 h. Total RNA was isolated from the cells using TRIzol reagent (Invitrogen Co., Carlsbad, CA, USA) and converted to cDNA using oligo(dT) primers (Intron Biotechnology Inc., Seongnam, Korea). For PCR amplification, the cDNA and specific primers (Table I) were added to 2X TOPsimple™ DyeMIX-nTaq (Enzynomics, Inc., Daejoen, Korea) and 0.1% diethylpyrocarbonate (DEPC) water. The amplified products were analyzed on 1% agarose gels stained with RedSafe™ nucleic acid staining solution (Intron Biotechnology, Inc.).

### Treatment with a MEK inhibitor (PD98059)

The MEK inhibitor, PD98059 was obtained from Cell Signaling Technology (Beverly, MA, USA) and stored as a 20 mM stock solution at −20°C. The cells were pre-treated with 40 *μ*M PD98059 for 1 h and then incubated for 24 h as described above.

### Cell cycle analysis

The cells were cultured in 6-well plates to 50–60% confluence and treated with SFM or various doses of PYP1 (1–20) (125, 250, 500 and 1,000 ng/ml) for 24 h. The cells were harvested after trypsinization, washed with PBS, and treated with cold PI solution (50 *μ*g/ml) containing RNase A (0.1 mg/ml) in PBS (pH 7.4) for 30 min in the dark. Flow cytometry was performed using a FACSCalibur instrument (Becton-Dickinson, San Jose, CA, USA).

### Statistical analysis

Multiple mean values were compared using analysis of variance with SPSS (SPSS Inc., Chicago, IL, USA). Values are the means ± SD. Different letters were used to indicate significant values according to Duncan’s multiple range test.

## Results

### PYP1 (1–20) increases the expression of EGFR and EGFR-related proteins

**Figure 1:**
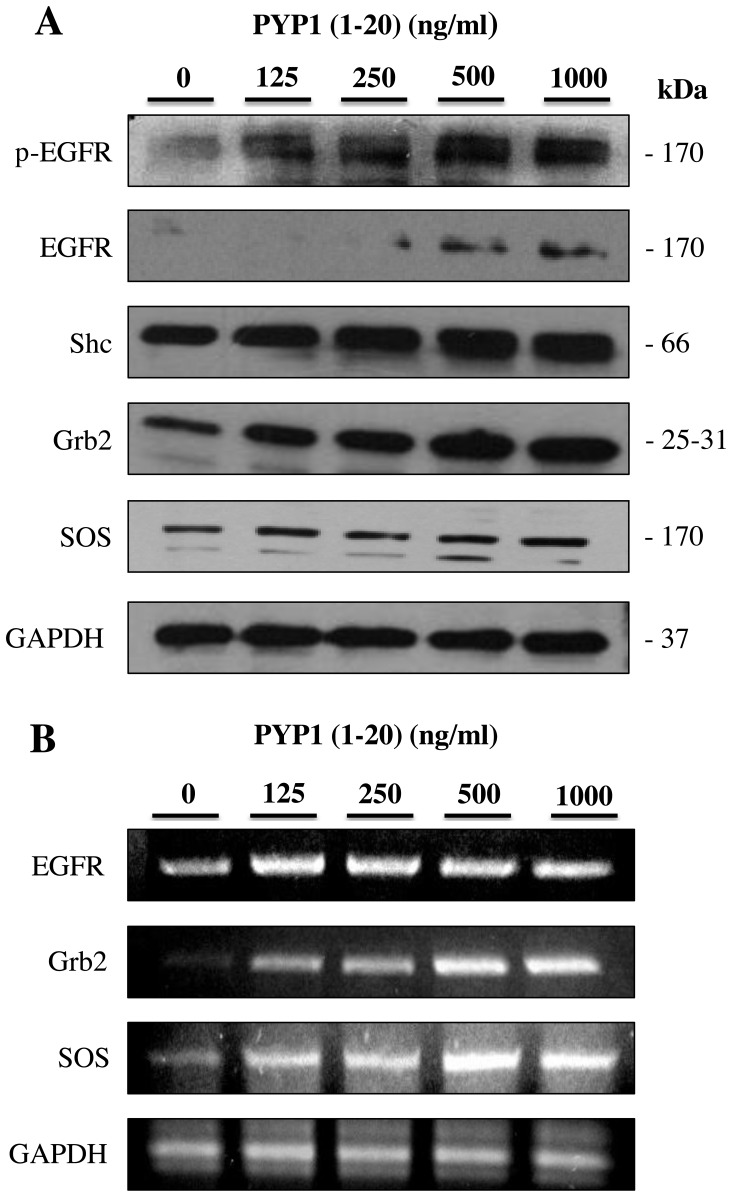
Effect of treatment with *Pyropia yezoensis* peptide [PYP1 (1–20)] on epidermal growth factor receptor (EGFR), Shc, growth factor receptor bound protein 2 (Grb2) and son of sevenless (SOS) protein and mRNA expression levels in IEC-6 cells. Proteins were subjected to western blot analysis and cDNA was subjected to RT-PCR. (A) Protein expression levels were increased upon incubation with PYP1 (1–20) for 24 h. (B) mRNA expression levels were also increased, as demonstrated by RT-PCR.

To investigate the mechanisms responsible for the PYP1 (1–20)-induced proliferation of IEC-6 cells, we examined the effects of PYP1 (1–20) on EGFR signaling-related proteins. The protein and mRNA expression levels of phoshorylated (p-)EGFR, Shc, Grb2 and SOS in the IEC-6 cells treated with PYP1 (1–20) (125, 250, 500 and 1,000 ng/ml) for 24 h were measured by western blot analysis and RT-PCR. Treatment with PYP1 (1–20) upregulated the protein (Fig. 1A) and mRNA (Fig. 1B) expression levels of p-EGFR, Shc, Grb2 and SOS in a dose-dependent manner. These results indicate that PYP1 (1–20) promotes the expression of EGFR signaling-related molecules.

### PYP1 (1–20) induces the activation of the Ras-p42/p44 MAPK signaling pathway

**Figure 2:**
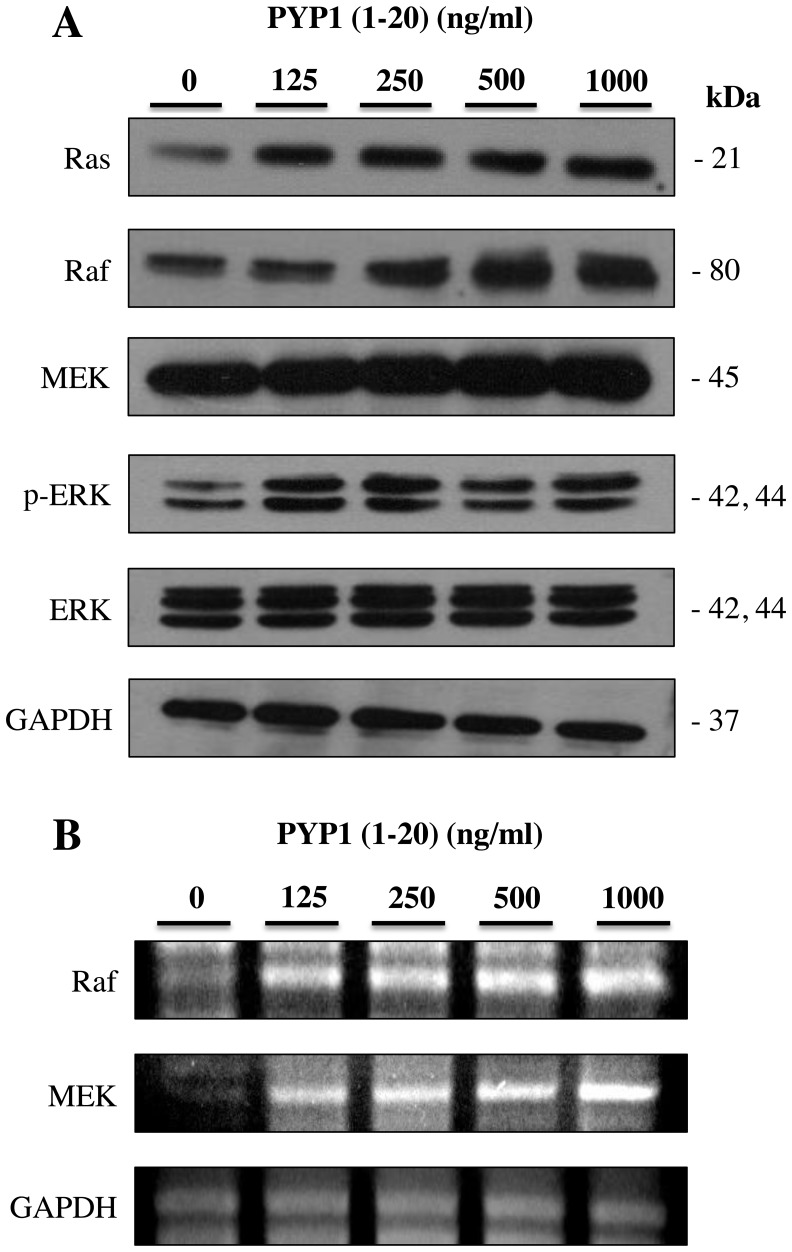
Effects of treatment with *Pyropia yezoensis* peptide [PYP1 (1–20)] on Ras, Raf, MEK and ERK (A) protein and (B) mRNA expression levels in IEC-6 cells. Whole cell extracts were prepared and analyzed by western blot analysis using anti-Ras, anti-Raf, anti-MEK, anti-phosphorylated-ERK, anti-ERK and anti-glyceraldehyde 3-phosphate dehydrogenase (GAPDH) antibodies.

To further determine the downstream signals regulated by EGFR activation, the protein and mRNA expression levels of the Ras-p42/p44 signaling pathway members were measured in the IEC-6 cells. The IEC-6 cells were treated with PYP1 (1–20) (125, 250, 500 and 1,000 ng/ml) for 24 h and then subjected to western blot analysis and RT-PCR. Treatment with PYP1 (1–20) for 24 h resulted in increased protein (Fig. 2A) and mRNA (Fig. 2B) expression levels of Ras, Raf, MEK and p-ERK compared with the untreated controls. These results indicate that PYP1 (1–20) activates the Ras-p42/p44 MAPK signaling pathway in IEC-6 cells.

### Pre-treatment with MEK inhibitor suppresses the PYP1 (1–20)-induced cell proliferation

**Figure 3:**
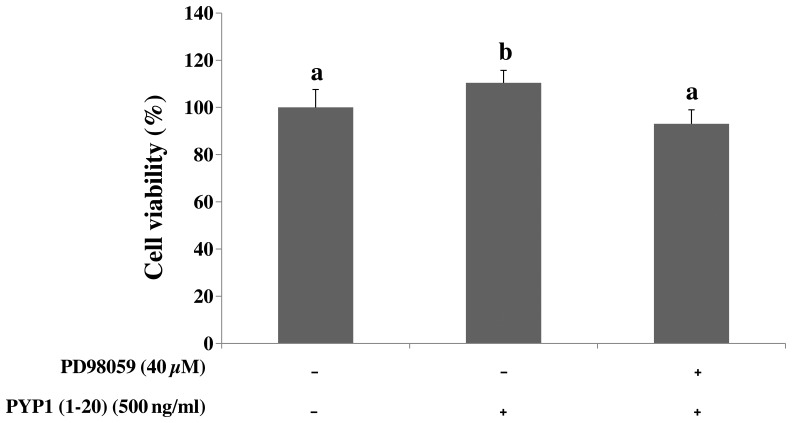
Effects of the MEK inhibitor (PD98059) on the *Pyropia yezoensis* peptide [PYP1 (1–20)]-induced proliferation of IEC-6 cells. Cells were seeded in 96-well plates at 1×10^4^ cells/well in 100 *μ*l medium. After 24 h, the cells were maintained in SFM for 4 h. Following pre-treatment with PD98059 (40 *μ*l), the cells were incubated with PYP1 (1–20) for 24 h. Cell viability was measured using an MTS assay kit as per the manufacturer’s instructions. Values represent the means ± SD. Different letters indicate significant values according to Duncan’s multiple range test.

To investigate the suppressive effect of the MEK inhibitor (PD98059) on PYP1 (1–20)-induced cell proliferation, an MTS assay was performed. Pre-treatment with PD98059 for 1 h, followed by the addition of PYP1 (1–20) (500 ng/ml) for 24 h, induced a decrease in cell viability identical to that of the controls (Fig. 3). Thus, EGFR is a target of PYP1 (1–20)-induced cell proliferation.

### Effect of PYP1 (1–20) on cell cycle progression

**Figure 4:**
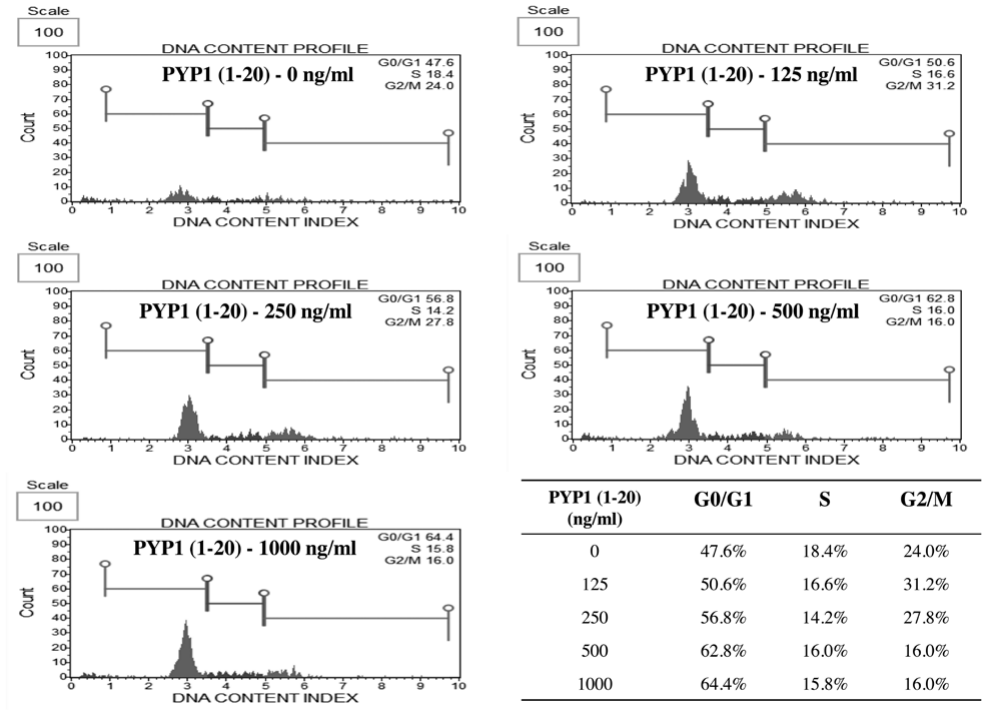
Treatment of IEC-6 cells with *Pyropia yezoensis* peptide [PYP1 (1–20)] resulted in cell cycle progression. In the dose dependence experiments, PYP1 (1–20) was added at varying concentrations (125, 250, 500 and 1,000 ng/ml).

The percentage of cells at each phase of the cell cycle was examined by flow cytometry. The cell cycle response was determined in the cells treated with various concentration (125, 250, 500 and 1,000 ng/ml) of PYP1 (1–20) for 24 h. Treatment with PYP1 (1–20) increased the percentage of IEC-6 cells in the G1 phase (47.6, 50.6, 56.8, 62.8 and 64.4% following treatment with 0, 125, 250, 500 and 1,000 ng/ml PP-YE, respectively) in a dose-dependent manner (Fig. 4). Therefore, treatment with PYP1 (1–20) markedly increased the proportion of cells in the G1 phase, suggesting that PYP1 (1–20) promotes IEC-6 cell cycle progression.

### Effect of PYP1 (1–20) on the expression of cell cycle-related proteins

**Figure 5:**
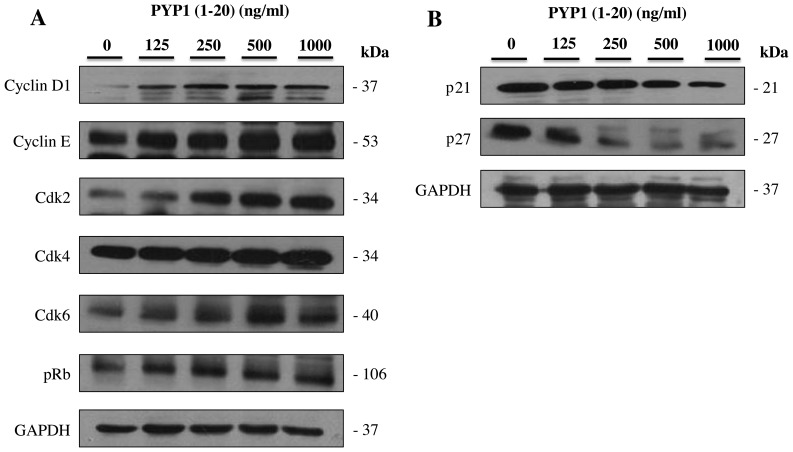
Effect of treatment with *Pyropia yezoensis* peptide [PYP1 (1–20)] on the levels of cell cycle-related proteins in IEC-6 cells. Cells were treated with PYP1 (1–20) after pre-incubation with SFM for 4 h. Whole cell extracts were prepared and analyzed by western blot analysis using (A) anti-cyclin D1, anti-cyclin E, anti-Cdk2, anti-Cdk4, anti-Cdk6 and anti-pRb; and (B) anti-p21, anti-p27 and anti-glyceraldehyde 3-phosphate dehydrogenase (GAPDH) antibodies.

The regulation of cell proliferation is defined as the increase in the cell number resulting from the completion of the cell cycle (23). To confirm the cell proliferation mechanisms through which PYP1 (1–20) promotes cell cycle progression, we examined the cell cycle-related protein content. The expression levels of cyclin D1, cyclin E, Cdk2, Cdk4, Cdk6, pRb, p21 and p27 were measured by western blot analysis using specific antibodies. The IEC-6 cell cycle response was determined following treatment with PYP1 (1–20) at various concentrations (125, 250, 500 and 1,000 ng/ml). The protein expression levels of cyclin D1, cyclin E, Cdk2, Cdk4, Cdk6 and pRb increased, whereas those of p21 and p27 decreased following treatment with PYP1 (1–20) for 24 h (Fig. 5). These results suggest that PYP1 (1–20) promotes IEC-6 cell proliferation by modulating the cell cycle-related proteins.

## Discussion

Many types of seaweed have received a great deal of attention from researchers in recent years for their high levels of nutrients, such as proteins, minerals, vitamins and polysaccharides. In particular, the anti-inflammatory and antitumor activities of seaweed have been studied extenstively (24,25).

In a previous study, we demonstrated that treatment with PYP1 (1–20) promotes the proliferation of IEC-6 cells through insulin-like growth factor I receptor (IGF-IR) signaling pathways (7). In the present study, we investigated whether PYP1 (1–20) promotes IEC-6 cell proliferation and cell cycle progression through the EGFR signaling pathway.

The activation of EGFR has been detected in many different cell types, such as epithelial, nerve and mesenchymal cells (14,15). EGFR activity induced by EGF binding has been implicated in essential cellular functions, including migration, differentiation, survival and proliferation (12,23). Activated EGFR leads to the activation of downstream signaling pathways, such as the Ras-p42/p44 MAPK pathway. The Ras-p42/p44 MAPK signaling cascade is a key mediator of growth factor-dependent cell survival, proliferation and differentiation (24). The activation of Ras occurs mostly via adaptor complex proteins containing Shc, Grb2 and SOS (16). In this study, PYP1 (1–20) increased the protein and mRNA expression levels of EGFR, Shc, Grb2 and SOS (Fig. 1). Therefore, we confirmed the effects of PYP1 (1–20) on the Ras-p42/p44 MAPK signaling pathway. Ras/Raf/MEK/ERK signaling commences at the cell surface, leading to the regulation of gene expression within the cell nucleus (6). In this study, in accordance with PYP1 (1–20)-induced cell proliferation, Ras, Raf, MEK and ERK, important mediators that regulate cell survival, growth and proliferation, were activated by exposure to PYP1 (1–20) (Fig. 2).

PYP1 (1–20)-induced cell proliferation was examined by cell cycle analysis (Fig. 4). Treatment with PYP1 (1–20) markedly increased the proportion of cells in the G0/G1 phase from 47.6 to 64.4%, suggesting that PYP1 (1–20) promotes cell cycle progression (Fig. 4). The PYP1 (1–20)-induced cell cycle progression resulted in cell proliferation and was related to the expression of cell cycle-related proteins, such as cyclin and Cdk. The expression levels of cyclin D1, cyclin E, Cdk2, Cdk4, Cdk6, pRb, p21 and p27 were measured by western blot analysis. The expression levels of cyclin D1, cyclin E, Cdk2, Cdk4, Cdk6 and pRb were increased in a dose-dependent manner, whereas the expression levels of p21 and p27 decreased in a dose-dependent manner (Fig. 5). Thus, the PYP1 (1–20)-induced cell cycle progression resulted in IEC-6 cell proliferation.

In the present study, we demonstrate that PYP1 (1–20) mediates cell proliferation through an EGFR signaling pathway in IEC-6 cells. These findings suggest the significant role of EGFR in intestinal epithelial cell proliferation, as well as the potential role of PYP1 (1–20) as a bio-functional food with a proliferative effect on rat intestinal epithelial cells.

## Figures and Tables

**Table I:** Oligonucleotide sequences of the primers used in RT-PCR.

Gene name	Primer sequences (5′→3′)
EGFR	F: CTC-ACG-CAG-TTG-GGC-ACT-TT R: TCA-TGG-GCA-GCT-CCT-TCA-GT
SOS1	F: GCA-TCT-TAT-TGG-AAG-GAT-TT R: CCT-CTC-AGG-TGA-GAC-TGC-TA
Grb2	F: CGG-GAT-CAT-GGA-AGC-CAT-GGC-CAA-A R: CTA-GCT-AGC-TTA-GAC-GTT-CCG-GTT-CAC-TG
Ras	F: CCC-GTC-CTC-ATG-TAC-TGG-TC R: ATC-TTG-GAT-ACG-GCA-GGT-CA
Raf	F: AAG-GCA-GTC-GTG-CAA-GCT-CA R: GAT-GAT-GGC-AAA-CTC-ACG-GAT-TG
MEK	F: CGA-TGG-ATC-CCC-CAA-GAA-GAA-GCC-GAC-G R: CGA-TCT-CGA-GTT-AGA-CGC-CAG-CAG-CAT-G
GAPDH	F: CAG-CCG-AGC-CAC-ATC-G R: TGA-GGC-TGT-TGT-CAT-ACT-TCT-C

RT-PCR, reverse transcription-polymerase chain reaction; EGFR, epidermal growth factor receptor; SOS1, son of sevenless; Grb2, growth factor receptor bound protein 2; GAPDH, glyceraldehyde 3-phosphate dehydrogenase; F, forward; R, reverse.
